# Validation of the Danish Version of Functional Assessment of Multiple Sclerosis: A Quality of Life Instrument

**DOI:** 10.1155/2011/121530

**Published:** 2011-10-30

**Authors:** Jan Sørensen, Jette Bay, Torben Damsgaard, Elsebeth Heeley, Ida Rostgaard, Brita Løvendahl, Finn Boesen

**Affiliations:** ^1^Center for Applied Health Services Research and Technology Assessment, University of Southern Denmark, J.B. Winsløws Vej 9B, 5000 Odense, Denmark; ^2^The Danish Multiple Sclerosis Society, Mosedalvej 15, 2500 Valby, Denmark; ^3^The Danish Multiple Sclerosis Hospital, Ringstedvej 106, 4690 Haslev, Denmark

## Abstract

The functional assessment of multiple sclerosis (FAMS) is a disease-specific instrument that describes functional status of individuals with multiple sclerosis. The instrument was originally developed in the US and has been adapted to different languages including Danish. This study is a validation of the Danish version of FAMS in a sample of individuals referred to a four-week rehabilitation program at either of the two Multiple Sclerosis Rehabilitation centers in Denmark. FAMS data were obtained through self-completed questionnaires from 190 individuals who attended the rehabilitation centers after referral by their general practitioner or specialist neurologist. The validation of the FAMS included assessment of data quality, scale assumptions, acceptability, construct validity, and reliability. Responsiveness was assessed by comparing individual FAMS scores at admission with the discharge score for groups of respondents who reported no change, improvement, or deterioration in their ability to cope with their illness. The Danish version of FAMS appears to be an acceptable, valid, and reliable measure of current health and functional status of individuals with multiple sclerosis.

## 1. Introduction

Multiple sclerosis is a chronic disease of the central nervous system. The Danish national registry of multiple sclerosis (MS) was started in 1956 [1]. The long history of this registry enables the analysis of a wide range of epidemiological studies, including analysis of the historical pattern of MS development and health consequences. A recent study estimated that 173 individuals per 100.000 population were affected by the disease, twice as many women as men [2]. The study found that the prevalence had increased three-fold since 1950, probably more due to environmental factors than genetic changes. Another study showed that the mean survival time of patients with MS is reduced by 12-13 years [3, 4], probably influenced by a suicide rate that is twice as high as in the general population [5, 6]. The social cost of Danish patients with MS is considerable and has been estimated to exceed 232 mio. € in 2005 [7].

Assessment of patients' health-related quality of life is important in clinical evaluations, as clinical assessments will not always reflect impact of illness and treatment on the patient's daily life [8]. MS can have significant impact on quality of life, for example, in the form of fatigue, pain, and mobility problems [9]. Application of quality of life instruments in clinical trials might offer important insight into patient's perceived outcomes. The effect of an experimental intervention might be a lower reduction in quality of life in comparison with the control intervention which might suggest that the experimental intervention is associated with more desirable health outcomes. A systematic review of quality of life studies in MS identified 19 disease-specific and 16 generic instruments that had been applied in 81 different studies [10]. However, none of the identified instruments fully satisfied all assessment criteria, and most offer a compromise between ease of use and comprehensiveness. The choice of instrument is thus partly dependent on the objectives of measurement [10]. Instruments suitable for clinical evaluations should fulfill a range of psychometric properties including reliability, validity, and responsiveness [11].

The functional assessment of multiple sclerosis (FAMS) is one of the disease-specific quality of life instruments available. It has been described as the best instrument for assessing quality of life of patients with MS as it covers many of the quality of life domains relevant to patients with MS and has shown good convergent validity [10, 12]. The aim of the current study was to validate the Danish version of the FAMS instrument using a sample of individuals who attended a long-term program at either of the two specialized Danish centers for MS rehabilitation. The psychometric properties of the FAMS were tested by assessing validity, reliability, and responsiveness to change.

## 2. Methods

### 2.1. Participants

The study sample consisted of individuals referred by general practitioner or specialist neurologist to a long-term rehabilitation program at the rehabilitation centers at Ry or Haslev and attended the centre between January 2007 and June 2008. The two centers are run by an independent organization that provides specialized medical and allied rehabilitation services to individuals with MS in line with agreements with regional health authorities. Together, the two centers have 78 beds and provide rehabilitation services to more than 1000 patients annually. Participants in this study were all admitted to a four-week standardized rehabilitation program for which there is a waiting time of 5–12 months after referral.

After giving informed consent, participants completed the FAMS at admission to the center. The data collection was approved by the Danish Data Protection Agency (ID no. 2009-41-3587). According to Danish regulations, studies collecting questionnaire data only do not require review by an ethics committee.

### 2.2. FAMS

The FAMS instrument was developed in Chicago, USA to be included in clinical trials and clinical processes. The development of the instrument [13] was based on a pool of 88 questions generated from interviews with patients and providers as well as review of the literature. Principal component analysis and Rasch modeling were applied to data from 377 patients with MS treated at two specialized hospitals, resulting in a reduction of the initial pool to 44 questions that were related to six domains: mobility, symptoms, emotional wellbeing (depression), general contentment, thinking/fatigue, and family/social wellbeing. In addition, 15 of the initial questions were retained due to potential clinical or empirical value. The final US version comprises 59 statements where the respondents are asked to indicate how true each statement has been for them during the past 7 days, using the following five categories: not at all, a little bit, and somewhat, quite a bit, and very much.

The scoring algorithm for FAMS assigns a value between 0 and 4 to each response category. The scores of negatively worded statements are reversed so, a high score consistently reflects good functional status/quality of life. The scores are added within each of the six subscales and then aggregated into a total FAMS score. One subscale (thinking/fatigue) has a range of 0–36, while the others have a range of 0–28. The total FAMS score ranges between 0 and 176. In the case of missing response items, a subscale score is derived based on the valid responses and adjusted so that the score maintains its full range.

The Danish version of FAMS had been previously translated and subsequently pilot tested among 90 MS patients undergoing in-hospital rehabilitation [14].

### 2.3. Supplementary Data Collection

Every Danish citizen has a unique personal identification number that is used in various national and local databases. This facilitated retrieval of the respondents' personal characteristics and enabled questionnaire data to be linked to data from other sources. In this study, clinical characteristics (type of MS, year of MS diagnosis, and Expanded Disability Status Scale (EDSS) score [15] at admission) were retrieved retrospectively from medical records by clinical experts and were supplemented by data from the local patient administrative systems.

### 2.4. Instrument Evaluation

This validation study was inspired by a validation study of the generic SF-36 instrument [16] and validation studies of other MS-specific quality of life instruments [12, 17, 18]. The FAMS instrument was evaluated using the following criteria.

**Data Quality**

It indicates the extent to which a scale can be used successfully in the target population and is determined by examining the percentage of missing data. The scoring procedure of FAMS includes a procedure for estimating scores despite missing response items. It is thus possible to derive scores for all scales, although the validity of the scores is compromised with missing response items. A low proportion of missing items is desirable, however.

**Scaling Assumptions**

They indicate whether it is legitimate to generate a total score by summing item scores without weighting or standardization. It is suggested that if individual items have item-subscale correlations exceeding 0.3, then item weighting might not be required, and if items have similar mean scores and variances, then standardization might not be required [19].

**Acceptability**

It concerns the extent to which the range of health measured by the scale matches the range of health in the sample. Ideally, scores should span the whole scale range, mean scores should be near the scale midpoint, floor/ceiling effects should be small (e.g., <15% of the sample), and skewness statistics should lie in the range of −1 to +1 [19].

**Reliability**

It concerns the extent to which the scale score is free from random error. It can be determined by inspection of Cronbach's alpha coefficient. When comparing patient groups, it is recommended that reliability should exceed 0.7, and precision in analyzing individual patient scores requires reliability exceeding 0.9 [16].

**Construct Validity**

It assesses the extent to which the instrument scores reflect expected relationships with other measures. It was measured here primarily by known-group comparisons, that is, patients with current active disease (primary or secondary progressive) could be expected to have worse scores than patients with remitting relapsing MS [13]. Older patients could also be expected to have lower scores. Another type of construct validity (convergent validity) was also to be tested by comparing the FAMS mobility subscale and the EDSS (ambulation) score.

**Responsiveness**

Was assessed by comparing the scores at admission and discharge. Respondents were categorized according to their reported change in satisfaction with the way they handle their illness (this is question 51 in the FAMS, but it is not used to derive subscale scores). As the rehabilitation program aims at supporting participants to cope with their illness, this question could be considered as a proxy indicator for the outcome of the rehabilitation intervention. Respondents were categorized according to the difference between admission and discharge scores as unchanged, better, or worse at discharge. Changes in score were tested with paired *t*-tests, where *P*  values < 0.05 were considered statistically significant. The effect size was calculated as the ratio of the change in the mean score and the baseline score standard deviation. Estimates of 0.2–0.5 indicate a small effect size, 0.5–0.8 a medium effect size, and over 0.8 a large effect size [19]. Comparison of score differences for respondents reporting better or worse state at discharge in comparison with unchanged state are reported as mean differences and 95% confidence intervals.

## 3. Results

Table 1 presents the sample characteristics. Of the 190 individuals who completed the FAMS at admission, six did not complete the FAMS at discharge. The 82 patients from the Ry center scored significantly lower on the FAMS total score than those from the Haslev centre (105 versus 115 *P* = 0.02), otherwise there were no significant differences between patients at the two centers. There were no statistical significant differences in total FAMS score according to gender (*P* = 0.84), 10-year age groups (*P* = 0.24), disease duration (0.51), type of MS (*P* = 0.42), or ambulation status (*P* = 0.32).

Table 2 presents results relating to data quality, scaling assumption, acceptance, and reliability. The proportion of missing items ranged from 5% to 11% percent in the six subscales. However, more than a quarter of the respondents did not provide valid response on at least one of the 44 questions. Two questions (Q41: “My family/close relatives have accepted my illness” and Q42: “I am satisfied with the way we talk about the illness in the family”) were left unanswered by 43% and 45% of the respondents, respectively, and two other questions (Q23: “I have accepted my illness” and Q24: “I am able to enjoy life”) were not answered by 22% and 26%, respectively. Other questions had nearly 100% completion (e.g., Q18: “I feel trapped by my condition” and Q37: “I have trouble learning new tasks or directions” each had <1% missing answers). It is worth noting that nearly all the questions in the mobility subscale had 10–12% missing responses.

Analysis of scaling assumptions showed that item-scale correlations for the six subscales ranged from 0.36 to 0.83, suggesting that a weighting of the items in the subscale is unnecessary. The score ranges and variations are broadly similar across subscales, such that standardization would not be required. The only exception is perhaps the mobility subscale, which had relatively low scores in comparison with the other subscales.

The analysis of acceptability showed that the reported scores covered a large section of the possible score ranges. Only a few patients had the minimum or maximum subscale scores, and no patients scored at the endpoints of the total FAMS score. The analysis of skewness provided negative values, reflecting relatively few low (poor health state) scores. Mobility was the only subscale with a positive skewness, reflecting a greater number of respondents with higher scores (i.e., fewer problems with mobility).

In terms of reliability, all subscales except general contentment reached the recommended level for group comparison (0.7), while not all the subscales reached the level required for patient-level comparisons (0.9). Cronbach's alpha for the total FAMS score was of 0.94, suggesting that the scale has sufficient reliability for patient-level comparisons.

Table 3 shows the analysis of construct validity. As could be expected, there were significant score differences between respondents with different types of MS, and older patients had lower (worse) mobility scores. Respondents at the two centers differed on the subscales for symptoms, thinking/fatigue, and family/social well-being, which was also reflected in the total FAMS score. This difference became insignificant after adjustment for patient characteristics such as age, gender, disease duration, and EDSS score (OLS regression, data not shown). There was a clear association between mobility subscale score, and ambulation status (EDSS score). The scores on the subscale thinking/fatigue differed according to disease duration, but the overall pattern was not clear as respondents with MS duration of 6–9 years had relatively high scores.

Table 4 presents analysis of FAMS score changes over time according to reported change in satisfaction with ability to handle the illness. The effect size for the patient group who gave the same score on Q51 at discharge as at admission (*n* = 111) was positive but relatively low, ranging from 0.05 to 0.20. The changes in subscales scores were modest although statistically significant for symptoms, general contentment, thinking/fatigue subscales, and the total FAMS score (all slightly better at discharge).

Respondents who at discharge reported greater satisfaction with ability to cope with the illness (Q51) also had statistically higher scores on all subscales including the total FAMS score. The effect size ranged from 0.24 to 0.57, indicating low to medium responsiveness to change. In comparison with the group that reported the same score on Q51 at discharge, these “improved” patients had statistically significant score differences on all scores except the symptoms and thinking/fatigue subscales.

Respondents who at discharge reported less satisfaction with ability to cope with the illness (Q51) also had consistently lower subscale scores and total FAMS score, although only the family/social well-being subscale reached statistical significance. The effect sizes were negative and ranged from −0.03 to −0.25, indicating low responsiveness to change.

## 4. Discussion

The aim of this study was to evaluate the psychometric properties of the Danish version of the FAMS instrument based on a sample of patients attending a four-week intervention program at two specialized centers for MS rehabilitation. The instrument showed adequate acceptability and reliability, and there was evidence for construct validity and responsiveness to change. There were a high number of missing responses on some items, but this was adjusted for in the scoring algorithm.

The results from the current study are very similar to the US validation study [13]. The scoring of the subscales was broadly similar, although patients in the current study had lower (worse) scores on mobility and higher (better) scores on emotional well-being and general contentment. The characteristics of the participants in the two studies were broadly similar, although there was a slightly higher proportion of women in the US sample. The mean total FAMS score was 108 (sd 28) in the current study and 111 (sd 27) in the US study. The reliability determined by Cronbach's alpha was also similar although the US study reported higher alpha values for the mobility and general contentment subscales. The findings relating to the scaling assumptions were also similar, although the US study reported higher item-subscale correlation for the mobility items. The US study provided stronger support for construct validity (ability to distinguish groups known to differ from one another), possibly due to a larger sample size (*n* = 377). The current study showed good convergent validity for the mobility subscale in a comparison against EDSS ambulation status.

The current study was based on a group of consecutive MS patients attending a four-week rehabilitation program after referral from a general practitioner or neurological specialist. Patients were referred to the rehabilitation centers for a variety of indications, including physical rehabilitation and treatment of neurological symptoms. As such, the study sample reflects typical MS patients referred for rehabilitation, and the results are therefore likely to be generalizable to other groups of MS patients attending routine rehabilitation services.

The patients attending the two rehabilitation centers had significantly different baseline scores on several subscales and the total FAMS scale. However, this difference became insignificant after adjustment for patient characteristics such as age, gender, disease duration, and EDSS score.

It is envisaged that the FAMS instrument would be useful in clinical studies assessing the impact of various medical and rehabilitation interventions in MS, in particular the effect of new medications. In cross-sectional analyses, the instrument could also contribute to knowledge about quality of life in different groups of MS patients. With its seven subscales, the FAMS instrument covers a broad range of aspects of quality of life that are relevant to MS patients. This allows the instrument to be used as a profile measure (looking at changes on specific aspects or domains of quality of life), while the total FAMS score enables an overall assessment of health state change.

A limitation of this validation study is that no other health status instruments or assessments were administered at the time of completion of the FAMS. This means that testing of convergent and discriminant validity was not possible, except for a limited analysis in which the FAMS mobility subscale was compared with the EDSS ambulation score. For lack of a better alternative, a proxy indicator for outcome of the intervention program—the patients' own perception of how satisfied they were with their ability to cope with the illness—was used. Given the limitations of this approach (satisfaction with coping may not correlate directly with, e.g., symptoms or emotional well-being), the instrument appeared to be sufficiently responsive to enable identification of changes in coping over time. Assessment of test-retest properties of the FAMS instrument (i.e., in patients whose functional health is not expected to change in any major way over time) was not possible in this study. We did not collect data on comorbidity in this study. This would be useful in future studies as patient functional status could be affected by other illnesses or conditions besides MS. 

## 5. Conclusion

Measurement of health outcomes is central for clinical practice and health policy planning. In a disease like MS—a chronic progressive disorder—outcomes that include the patient's own perspective are important. The Danish version of FAMS appears to be an acceptable, valid and reliable measure of the current health and functional status. Future studies should assess the test-retest properties of FAMS and how well FAMS correlates with other disease-specific instruments such as the Multiple Sclerosis Impact Scale (MSIS-29) and with generic health status measures such as the SF-36/SF-12, EQ-5D, and 15D instruments. The results from this study suggest that the FAMS is appropriate for use in clinical trials to evaluate the effect of medical and rehabilitation interventions on patients' physical and psychosocial functioning.

## Figures and Tables

**Table 1:** Characteristics of the study participants (*n* (%)).

	Haslev (*n* = 108)	Ry (*n* = 82)	All (*n* = 190)
Gender:			
(i) men	42 (39%)	35 (43%)	77 (41%)
(ii) women	66 (61%)	47 (57%)	113 (59%)

Age group:			
(i) under 40 years	12 (11%)	6 (7%)	18 (9%)
(ii) 40–49 years	31 (19%)	27 (33%)	58 (31%)
(iii) 50–59 years	30 (28%)	26 (32%)	56 (29%)
(iv) over 60 years	35 (32%)	23 (28%)	58 (31%)

Disease duration:			
(i) 2 years or less	9 (8%)	13 (16%)	22 (12%)
(ii) 3–5 years	20 (18%)	9 (11%)	29 (15%)
(iii) 6–9 years	21 (19%)	21 (26%)	42 (22%)
(iv) 10–19 years	31 (29%)	29 (35%)	60 (32%)
(v) 20 years or more	25 (23%)	10 (12%)	35 (18%)
(vi) missing	2 (2%)	0 (0%)	2 (1%)

Type of MS:			
(i) relapsing remitting	28 (26%)	28 (34%)	56 (29%)
(ii) secondary progressive	56 (52%)	42 (51%)	98 (52%)
(iii) primary progressive	22 (20%)	12 (15%)	34 (18%)
(iv) missing	2 (2%)	0 (0%)	2 (1%)

Ambulation status:			
(i) walking independently (EDSS < 5.5)	42 (39%)	30 (37%)	72 (38%)
(ii) walking with aid (EDSS 6.0–6.5)	57 (53%)	38 (46%)	95 (50%)
(iii) wheelchair user (EDSS > 7.0)	8 (7%)	14 (17%)	22 (12%)
(iv) missing	1 (1%)	0 (0%)	1 (1%)

EDSS = Expanded Disability Status Scale [15].

**Table 2:** FAMS subscale scores and total score: data quality, scaling assumption, acceptability, and reliability (*n* = 190).

Psychometric property	Mobility (7 items)	Symptoms (7 items)	Emotional well-being (7 items)	General contentment (7 items)	Thinking/ fatique (9 items)	Family/social well-being (7 items)	FAMS total (44 items)
Data quality							
(i) Responses with missing items (%)	11.0	5.8	7.4	7.4	6.3	5.3	26.8

Scale assumptions							
(i) Subscore mean (range)	12.6 (1–27)	20.9 (6–28)	19.4 (1–28)	17.8 (0–28)	19.6 (0–35)	20.7 (5–28)	110.7 (34–167)
(ii) Item mean score	1.3–2.2	2.5–3.8	2.0–3.3	2.0–2.8	1.8–2.5	2.6–3.3	1.4–3.8
(iii) Item sd score	0.9–1.2	0.6–1.3	1.0–1.3	1.1–1.3	1.2–1.3	1.0–1.2	0.6–1.3
(iv) Item-scale correlation	0.53–0.77	0.36–0.79	0.65–0.84	0.63–0.83	0.66–0.81	0.68–0.80	0.10–0.77

Acceptability							
(i) Possible score range	0–28	0–28	0–28	0–28	0–36	0–28	0–168
(ii) Observed score range	1–27	6–28	1–28	0–28	0–35	5–28	34–167
(iii) Mean score (SD)	12.6 (5.2)	20.9 (5.2)	19.4 (6.4)	17.8 (5.9)	19.6 (8.5)	20.7 (5.6)	110.7 (28.0)
(iv) Median (IQR)	12 (9–16)	22 (18–25)	21 (15–24)	18 (13–22)	20 (13–27)	22 (17–25)	114 (91–131)
(v) Floor/ceiling effects (%)	0/0	0/7	0/6	1/2	1/0	0/8	0/0
(vi) Skewness	0.35	−0.73	−0.81	−0.35	−0.75	−0.75	−0.39

Reliability							
(i) Cronbach's alpha	0.79	0.79	0.90	0.62	0.91	0.85	0.94

**Table 3:** Construct validity of FAMS subscale scores and total score.

Characteristics of the respondents	Mobility	Symptoms	Emotional well-being	General contentment	Thinking/ fatique	Family/social well-being	FAMS total
Centre:							
(i) Haslev (*n* = 108)	13.3 (5.2)	21.7 (4.8)*	20.0 (6.2)	17.9 (5.8)	20.9 (7.9)*	21.4 (5.2)*	114.8 (26.3)*
(ii) Ry (*n* = 82)	11.7 (5.0)	19.9 (5.6)*	18.6 (6.7)	17.6 (6.2)	17.9 (8.9)*	19.7 (6.1)*	105.3 (29.4)*

Gender:							
(i) Men (*n* = 77)	11.9 (4.5)	21.3 (4.8)	19.8 (5.6)	17.8 (5.5)	19.0 (8.7)	21.4 (4.9)	111.2 (26.3)
(ii) Women (*n* = 113)	13.1 (5.6)	20.7 (5.5)	19.1 (6.9)	17.7 (6.2)	20.0 (8.3)	20.1 (6.0)	110.4 (29.2)

Age group:							
(i) Under 40 years (*n* = 18)	14.9 (4.7)*	22.8 (3.6)	21.5 (5.3)	19.5 (4.7)	21.1 (8.0)	19.5 (6.7)	120.0 (24.7)
(ii) 40–49 years (*n* = 58)	13.4 (5.9)*	20.4 (5.6)	18.9 (7.0)	17.3 (6.7)	18.5 (8.3)	19.6 (6.1)	108.0 (30.0)
(iii) 50–59 years (*n* = 56)	11.5 (4.9)*	30.4 (4.7)	18.7 (6.7)	16.9 (5.7)	19.0 (8.6)	50.7 (5.1)	107.1 (27.2)
(iv) Over 60 years (*n* = 58)	12.3 (4.6)*	21.5 (5.6)	20.0 (5.8)	18.5 (5.6)	20.5 (8.6)	22.0 (5.1)	113.9 (27.3)

Disease duration:							
(i) 2 years or less (*n* = 22)	12.5 (4.9)	20.3 (5.6)	18.8 (6.8)	16.5 (5.1)	18.0 (10.1)*	21.2 (5.1)	107.2 (30.0)
(ii) 3–5 years (*n* = 29)	13.5 (5.7)	21.1 (4.6)	19.1 (5.4)	16.6 (5.1)	18.3 (6.7)*	18.9 (5.2)	107.4 (24.4)
(iii) 6–9 years (*n* = 41)	13.1 (5.5)	21.3 (5.7)	20.4 (6.1)	19.0 (5.6)	22.9 (7.6)*	20.4 (6.1)	116.0 (27.1)
(iv) 10–19 years (*n* = 60)	12.4 (5.2)	20.3 (5.3)	19.5 (6.4)	17.6 (6.4)	18.0 (9.2)*	20.6 (5.7)	108.2 (28.9)
(v) 20 years or more (*n* = 35)	12.3 (4.6)	22.2 (4.3)	18.9 (7.6)	18.5 (6.4)	20.4 (7.8)*	22.3 (5.5)	114.5 (29.5)

Type of MS:							
(i) Relapsing remitting (*n* = 56)	14.9 (5.6)*	21.2 (5.4)	20.7 (6.5)	18.3 (5.8)	20.2 (9.6)	19.6 (6.1)	114.7 (30.4)
(ii) Secondary progressive (*n* = 98)	11.5 (4.8)*	20.6 (5.0)	18.8 (6.5)	17.4 (6.4)	19.1 (8.1)	21.3 (5.5)	108.5 (27.4)
(iii) Primary progressive (*n* = 34)	12.4 (3.9)*	21.8 (5.1)	19.2 (6.2)	18.1 (5.0)	20.3 (7.8)	20.7 (5.2)	111.4 (25.9)

Ambulation status:							
(i) Walking independently (EDSS < 5.5) (*n* = 72)	15.1 (5.2)*	20.7 (5.1)	20.0 (5.9)	18.2 (5.4)	19.3 (8.7)	19.6 (5.4)	112.8 (25.9)
(ii) Walking with aid (EDSS 6.0–6.5) (*n* = 95)	11.8 (4.5)*	21.0 (5.3)	19.5 (6.4)	17.9 (5.8)	20.0 (8.4)	21.9 (5.3)	111.6 (28.4)
(iii) Wheelchair user (EDSS > 7.0) (*n* = 22)	8.6 (4.1)*	21.2 (5.4)	17.3 (8.1)	15.9 (8.0)	19.0 (8.4)	18.7 (6.8)	100.6 (32.5)

*Indicates statistically significant difference (*P* < 0.05).

**Table 4:** Responsiveness to change: mean (sd) FAMS scores at admission and discharge for individuals who had same, better, or worse score at discharge on question 51: “Satisfaction with the way I handle my illness.”

Satisfaction with the way I handle my illness (Q51)	Mobility	Symptoms	Emotional well-being	General contentment	Thinking/ fatique	Family/social well-being	FAMS total
Same score (*n* = 111):							
(i) Score at admission	13.1 (5.7)	21.3 (4.9)	20.6 (5.9)	18.6 (5.8)	21.2 (7.8)	22.0 (5.0)	116.4 (27.0)
(ii) Change at discharge	0.8 (4.2)	0.9 (3.4)*	0.7 (4.1)	1.1 (4.1)*	1.6 (5.0)*	−0.3 (3.1)	4.8 (16.9)*
(iii) Effect size	0.15	0.19	0.12	0.18	0.20	−0.05	0.18

Better score (*n* = 43):							
(i) Score at admission	12.7 (5.0)	21.0 (5.2)	18.4 (6.0)	16.8 (5.6)	18.5 (8.3)	19.0 (6.0)	106.3 (24.7)
(ii) Change at discharge	2.3 (4.0)*	1.5 (4.0)*	2.9 (4.5)*	2.5 (3.9)*	3.3 (7.5)*	1.5 (3.8)*	14.0 (16.8)*
(iii) Effect size	0.44	0.29	0.48	0.45	0.40	0.24	0.57
Mean dif. from “same score” group							
(i) Mean (95% CI)	1.5 (0.1; 3.0)*	0.6 (−0.7; 1.8)	2.2 (0.7; 3.7)*	1.5 (0.0; 2.9)*	1.7 (−0.4; 3.7)	1.7 (0.6; 2.9)*	9.2 (3.0; 15.2)*

Worse score (*n* = 26):							
(i) Score at admission	11.5 (5.0)	19.7 (5.4)	17.4 (7.0)	16.0 (5.9)	16.7 (9.6)	17.6 (5.8)	97.8 (29.8)
(ii) Change at discharge	−0.4 (5.6)	−0.2 (2.9)	−0.9 (5.1)	−0.7 (4.0)	−1.2 (6.8)	−1.2 (2.7)*	−7.3 (23.1)
(iii) Effect size	−0.09	−0.03	−0.13	−0.12	−0.12	−0.21	−0.25
Mean dif. from“same score”group							
(i) Mean (95% CI)	−1.2 (−3.2; 0.7)	1.1 (−2.5; 0.3)	−1.6 (−3.5; 0.3)	−1.8 (−3.5; 0)*	−2.8 (−5.1;−.04)*	−1.0 (−2.3; 0.4)	−12.1 (−20.0;−4.3)*

*Indicates statistically significant difference (*P* < 0.05).
